# Thalidomide Prevented and Ameliorated Pathogenesis of Crohn’s Disease in Mice *via* Regulation of Inflammatory Response and Fibrosis

**DOI:** 10.3389/fphar.2019.01486

**Published:** 2019-12-13

**Authors:** Hongjin Chen, Haixia Xu, Lijiao Luo, Lichao Qiao, Yaohui Wang, Minmin Xu, Youran Li, Ping Zhu, Bolin Yang

**Affiliations:** ^1^Department of Colorectal Surgery, Jiangsu Province Hospital of Chinese Medicine, Nanjing, China; ^2^First Clinical Medical College, Affiliated Hospital of Nanjing University of Chinese Medicine, Nanjing, China; ^3^Department of Pathology, Jiangsu Province Hospital of Chinese Medicine, Nanjing, China

**Keywords:** Crohn’s disease, thalidomide, fibrosis, animal model, inflammation, mechanism

## Abstract

Crohn’s disease (CD) is a chronic, relapsing form of inflammatory bowel disease, seriously threatening human health. Thalidomide has been used for the treatment of CD. However, the effects and the possible mechanisms of thalidomide on CD are still unclear. Herein, our study demonstrated that thalidomide protected colon mucosa against trinitro-benzene-sulfonic acid (TNBS)-induced injury, diminished inflammatory infiltration and levels of IFN-γ, IGF-1, IL-6, IL-17, TNF-α, while increased the levels of IL-10 and TGF-γ. Moreover, it reversed the intestinal fibrosis and inhibited the accumulated infiltration, down-regulated the expression of col1a2, col3a2, MMP-3, MMP-9, MMP-1, TGF-γ, α-SMA, but up-regulated the expression of TIMP-1 and Vimentin. Although it could be observed that the effect of thalidomide administration in modeling was better than after modeling, there was no statistical difference between the two groups. The present study provided evidence that the therapeutic effect of thalidomide alleviated the inflammatory response and damage of colon tissue, mainly by restoring the imbalance of TH17/Treg cells and inhibiting intestinal fibrosis in TNBS-induced mice colitis.

## Introduction

Crohn’s disease (CD) is a chronic inflammatory granulomatous disease involving the whole digestive tract, and predominantly occurs at the end of the ileum and adjacent colon (Khanna and Raffals, 2017; Torres et al., 2017). The pathological changes of CD show a periodic and jumping distribution, and its pathogenesis may be closely related to heredity, immunity, environment, bacterial infection, mucosal regulation and so on (Kaistha and Levine, 2014; Mydock-McGrane et al., 2017; Rosen et al., 2017). CD is common in Europe and the United States with an annual incidence of 10.7/100, 000-20.2/100, 000, while the incidence of CD in Asia is relatively low. However, in recent years, studies have shown that the incidence of CD in China is increasing continuously and has become one of the common diseases (Ooi et al., 2016; Lai et al., 2019). The main clinical manifestations of CD include abdominal pain, diarrhea, abdominal mass, fistula formation, perianal lesions and protracted course, which seriously affects the living quality (Gajendran et al., 2018). Therefore, how to induce disease remission, prevent complications and improve the living quality is a hot issue that every clinician needs to be paid attention to.

Drugs for treating CD include aminosalicylic acid preparations, corticosteroids, immunosuppressive agents and biological agents (Zalieckas, 2017; Pudipeddi et al., 2019). Aminosalicylic acids are mainly used for remission of CD in mild active stage and corticosteroid for moderate or severe CD or patients who are ineffective with aminosalicylic acid, immunosuppressive agents for the maintenance therapy and biological agents are considered as redemption for patients who have failed in traditional treatment (Akobeng et al., 2016; Rutgeerts et al., 2004; Lémann et al., 2006; Ng et al., 2011; Zheng et al., 2011; Yang et al., 2012). However, the occurrence of steroid dependence and resistance or intolerance to medical therapy is still quite common. About 20-30% of patients experience primary clinical failure to biological agents while 30-40% of patients experience loss of response over time (Simon et al., 2016).

Thalidomide was initially marked as a sedative and antiemetic, mainly to control early pregnancy response (McBride, 1991). In 1965, Sheskin first published a series of studies on the successful treatment of erythema nodules of leprosy with thalidomide, suggesting that thalidomide has anti-inflammatory activity (De las Aguas, 1971). Thalidomide is emerging as an alternative treatment of selected patients with refractory CD and widely used in clinic (Hu et al., 2016; He et al., 2017; Wang et al., 2017; Zhu et al., 2017). Thalidomide could promote endoscopic and histologic healing in children with inflammatory bowel disease and induce clinical remission and mucosal healing in adults with active refractory CD (Simon et al., 2016; Lazzerini et al., 2017). Studies have shown that the thalidomide treatment reduce TNF-α, IL-6 and IL-1β production, and histological analysis showed considerable reduction in neutrophil infiltration and mucosal damage in colon homogenate of experimental colitis (Fakhoury et al., 2014). However, the cytokine suppressive properties and therapeutic potential of thalidomide in CD should be further investigated. In this study, thalidomide was used in the treatment of TNBS-induced mice colitis to clarify the therapeutic effects, and underlying mechanisms, so as to provide a theoretical basis of thalidomide to control the symptoms of active CD.

## Materials and Methods

### Experimental Animals

Sixty BALB/c mice weighting 16-20 g were obtained from Animal Experimental Center of Nanjing Medical University (Nanjing, China). All animals were housed under standard environmental conditions at controlled temperature (22 ± 2°C), humidity (50 ± 10%), and light (12 h light/dark cycle) with free access to standard diet and water. All procedures for animal care and use were in accordance with the National Institute of Health (NIH) guidelines, and approved by the Institutional Animal Care and Use Committee of Jiangsu Province Hospital of Chinese Medicine and Affiliated Hospital of Nanjing University of Chinese Medicine (2016NL-033-03).

### Induction of CD and Drug Administration

The CD-like colitis model was induced with TNBS as described previously (Tomasello et al., 2015). A total of 60 mice were randomized into three groups, listed as 45% ethanol group of 12 mice, saline group of 12 mice, and TNBS-induced groups of 36 mice. Briefly, mice were fasted overnight and lightly anesthetized with ether. Then, the animals were anesthetized, and 0.5 ml of 45% ethanol (sham-1), saline (sham-2) or TNBS (50 mg/kg) dissolved in 0.5 ml of 45% ethanol were instilled into the colon through a rubber catheter. The experiment was repeated weekly for four weeks.

CD-like TNBS-induced colitis mice were divided into three groups, model group, thalidomide in modeling group (thalidomide treatment was started at the beginning of TNBS induction), and thalidomide after modeling group (thalidomide treatment after TNBS induction). Thalidomide (Changzhou Pahrmaceutical Factory, Changzhou, China) (200 mg/kg, suspended in 0.5 ml olive oil) (Mazzon and Cuzzocrea, 2006) administered once daily *via* intragastric instillation every day for 4 weeks. Groups of sham-1, sham-2 and model received the vehicle (0.5 ml olive oil) by the same route.

### Assessment of Colonic Damage

The weight of mice was recorded daily, and the disease activity index (DAI) of the mice from different groups was evaluated daily according to criteria (Rachmilewitz et al., 2002). After intervention, the mice from each group were weighted and euthanized. Then the distal colon was carefully excised, weighted and measured about the length. The colonic samples were taken at 3 to 5 cm from the colon to the anal edge (1 cm in length) for further study.

### Hematoxylin-Eosin Staining

The colonic samples were fixed in 10% formalin, embedded in paraffin, and sequential serial sections were obtained. The sections were stained with hematoxylin-eosin (HE) staining. Ten random areas were examined in each section and identified by computer generated field identification. At least six different sections of colonic tissues were examined for each animal. Images were obtained using a fluorescence microscope (Nikon 80i).

### Masson Staining and Verhoeff’s Van Gieson (VEG) Staining

Isolated colonic tissues were fixed in 4% neutral formalin and embedded in paraffin. Then the sections (5 µm) were stained with Masson trichrome solutions. Images were obtained using a light microscope (Nikon 80i).

Isolated colonic tissue sections were stained in the Verhoeff’s staining solution for 30 min, differentiated in 2% ferric chloride (Sigma-Aldrich) for 1 min, rinsed briefly in running tap water and checked microscopically for black, sharply defined elastin staining. Slides were returned to 2% ferric chloride and microscopically checked again at 10-20 s intervals until the background appeared pale violet, and the vessels of interest remained sharply defined. The sections were treated with 5% sodium thiosulfate for 1 min and rinsed in running tap water for 5 min. The 1% light green solution was diluted to 0.5%. Slides were stained in this solution for 1 min and rinsed in running tap water for 30 s, dehydrated and cleared through graded alcohols and xylene, and coverslipped.

### Immunohistochemical Analysis

Isolated colonic tissues from different groups were stained for MMP-3 (ab52915), TIMP-1 (ab86482), E-cadherin (ab76055), N-cadherin (ab202030), Vimentin (ab193555), a-SMA (ab32575) and MMP-9 (ab38898). In briefly, isolated colonic tissues were fixed in 4% neutral formalin for 24 h, embedded in paraffin and were serially sectioned at 5 εm. Sections were deparaffinized and rehydrated, then submerged in hydrogen peroxide to quench peroxidase activity following incubation with 1% BSA to block non-specific binding sites. After incubation with primary antibodies at 4°C for 12 h, secondary antibodies were applied to slides for 1 h at room temperature. All the sections were visualized using diaminobenzidine (DAB, Beyotime) under a light microscope (Nikon 80i). Antibodies in immunohistochemical analysis were purchased from Abcam (Cambridge, MA, USA).

### Enzyme-Linked Immunosorbent Assay (ELISA)

The concentrations of cytokines in isolated colonic tissues were determine by enzyme-linked immunosorbent assay (ELISA) for mouse TNF-α, IL-6, IL-10, IL-17, TGF-β, IGF-1 and IFN-γ (eBioscience, San Diego, CA) following the manufacturer’s instructions.

### RNA Isolation and Quantitative Real-Time RT-PCR (qRT-PCR)

Total RNA was extracted from colonic tissues using TRIzol reagent (Invitrogen, USA) according to the manufacturer’s instructions. Total RNA (1 µg) was reversed and transcribed into cDNA using RNeasy plus micro kit. The total cDNA was used as starting materials for real-time PCR with FastStart Universal SYBR Green Master (Roche Applied Science, Mannheim, Germany) on the Step One real-time PCR System (Life Technologies Corp). The real-time PCR reactions were performed in triplicate.

### Western Blot Analysis

Total protein of colonic tissues was extracted according to the manufacturer’s protocol (Vazyme, USA). Briefly, protein concentrations were determined through BCA Protein Assay Kit (Vazyme, USA). Samples with equal amounts of protein (25 µg) were fractionated on 10% SDS polyacrylamide gels, transferred to polyvinylidene difluoride (PVDF) membranes, and blocked in 5% skim milk in TBST for 1.5 h at 25°C. The membranes were then incubated at 4°C overnight with 1:1,000 dilutions (v/v) of the primary antibodies. After washing the membranes with TBST, the secondary antibodies with 1:1,000 dilutions were added and incubated for another 2 h at 25°C. Protein expressions were examined using an Enhanced Chemiluminescence Detection System. GAPDH was used as a loading control. Antibodies in western blotting were purchased from Abcam (Cambridge, MA, USA), including Col1a2 (ab96723), Col3a2 (ab196613), MMP-1 (ab52631), MMP-3 (ab52915), TIMP-1 (ab86482), E-cadherin (ab76055), N-cadherin (ab202030), Vimentin (ab193555), a-SMA (ab32575), MMP-9 (ab38898) and GAPDH (ab181602).

### Statistical Analysis

Experimental data were analyzed with SPSS 21.0 software (SPSS Inc., Wacker Drive, Chicago, IL, USA) and Graphpad Prism 5 (Graphpad Software, San Diego, CA). All data are presented as the mean ± standard deviation (SD). Statistics among each experimental group were analyzed using one-way analysis of variance (ANOVA) followed by the least significant difference test. The level of significance was set at *p < 0.05, **p < 0.01 (compared with sham-1 and sham-2 groups); ^#^p < 0.05, ^##^p < 0.01 (compared with the model group).

## Results

### Effects of Thalidomide on the Body Weight and DAI in Mice Induced With TNBS

**Figure 1:**
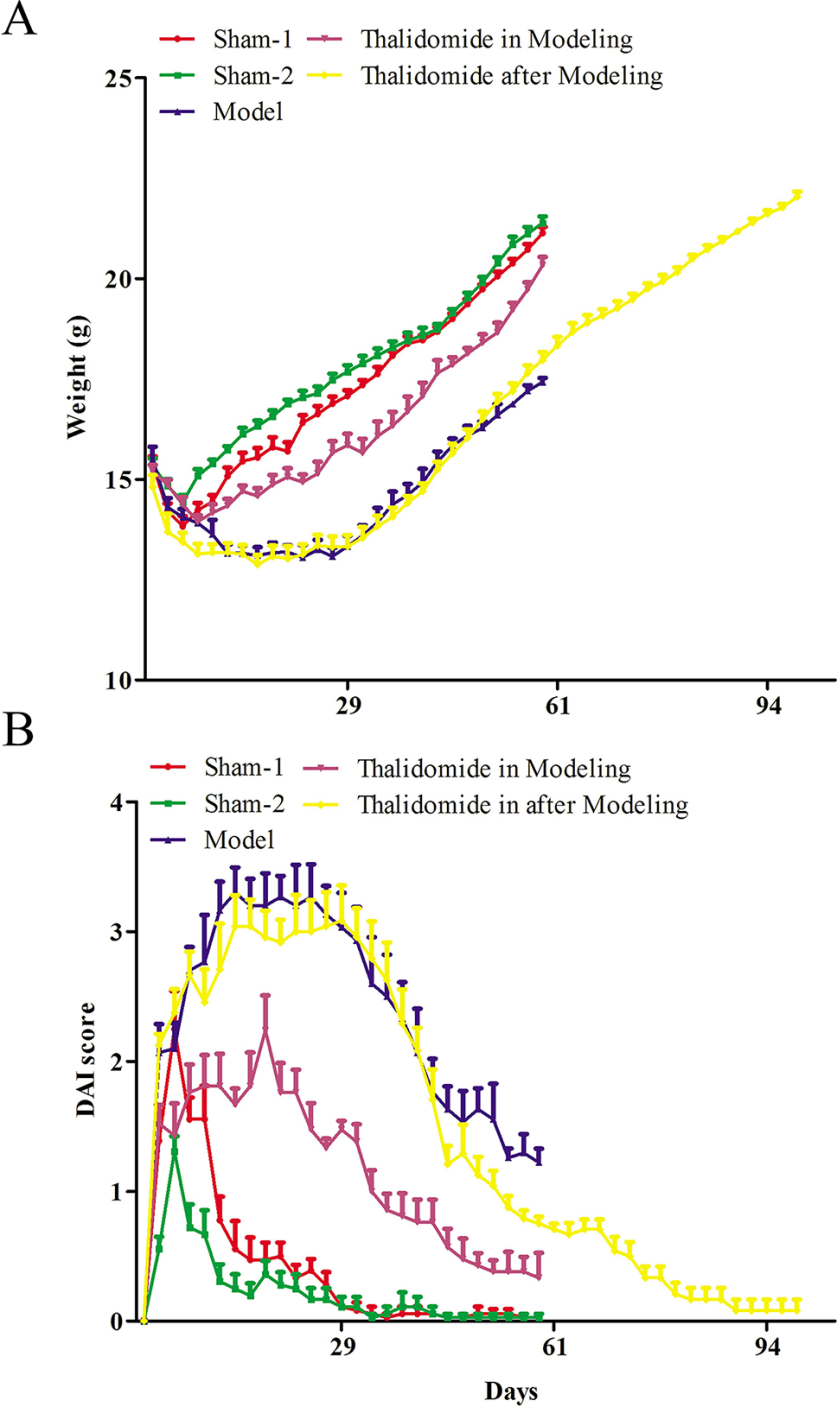
Effects of thalidomide on the body weight and DAI in mice induced with TNBS. **(A)** The body weight of mice from different groups. **(B)** The DAI score of mice from different groups. The results were expressed as the mean ± SD.

Firstly, to evaluate whether thalidomide has preventive and the therapeutic effects on the mice induced with TNBS, the body weight of TNBS-induced mice was measured. As indicated in **Figure 1A**, compared with sham-1 and sham-2 groups, TNBS treatment could decrease mice body weight, while treatment with thalidomide in modeling or after modeling could increase the body weight. Moreover, mice from different groups also had intense inflammatory response characterized by DAI. Treatment with thalidomide in modeling or after modeling could protect the colon against TNBS-induced injury compared with that in model group (**Figure 1B**).

### Effects of Thalidomide on the Colonic Weight and Length in Mice Induced With TNBS

**Figure 2:**
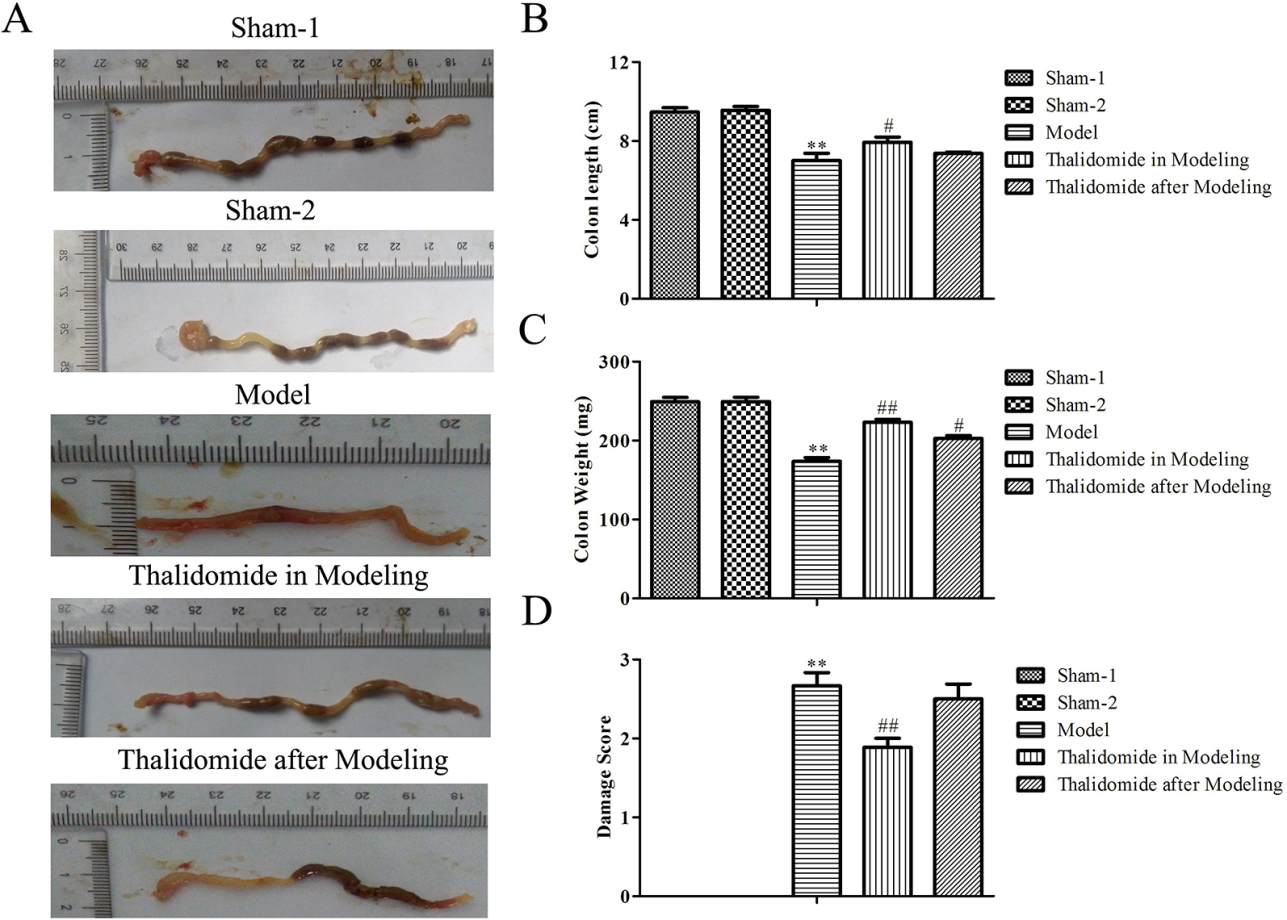
Effects of thalidomide on the colonic weight and length in mice induced with TNBS. The colonic length and weight of mice from different groups were measured, and treatment with thalidomide showed remarkably effects in TNBS-induced alterations in colonic weight, length and macroscopic scores. The results were expressed as the mean ± SD. **P < 0.01 compared with the sham-1 and sham-2 groups; ^#^ *P < *0.05, ^##^ *P < *0.01 compared with the model group. **(A)** Macroscopic appearance of colons from each group. **(B)** Colon length of each group. **(C)** Colon weight of each group. **(D)** Macroscopic score of each group.

The colonic weight and length of mice were measured. As shown in **Figures 2A-D**, compared with the sham-1 and sham-2 groups, there was significant decrease in colonic weight and length and obviously increase in macroscopic scores in model group, respectively. Interestingly, compared with model group, treatment with thalidomide in modeling or after modeling showed remarkably effects in TNBS-induced alterations in colonic weight, length and macroscopic scores.

### Effects of Thalidomide on the Colonic Histological Alteration in Mice Induced With TNBS

**Figure 3:**
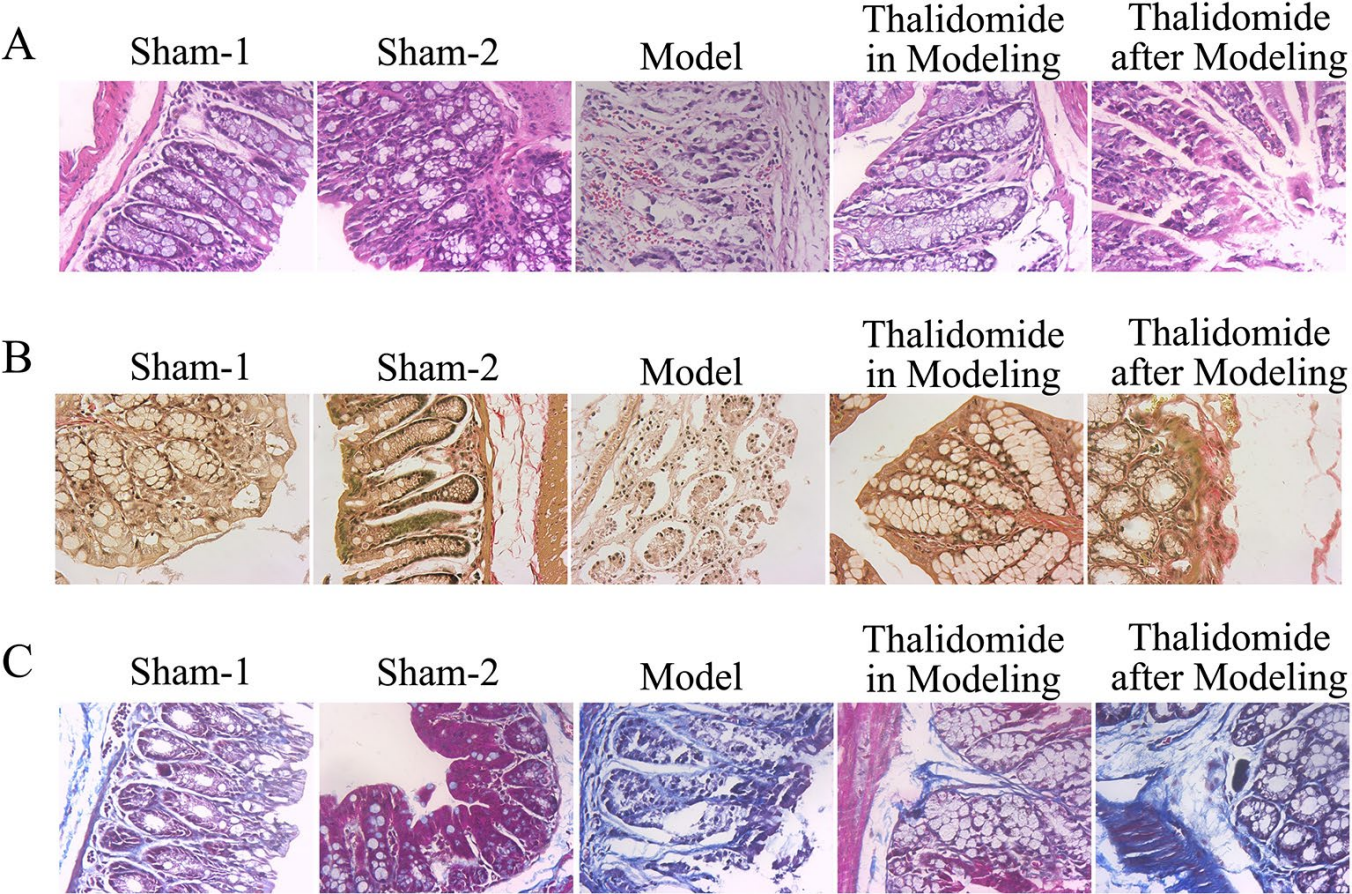
Effects of thalidomide on the colonic histological alteration in mice induced with TNBS. **(A)** The severity of colonic inflammation of mice from different groups was evaluated by HE staining. Thalidomide treatment decreased the inflammatory infiltration induced by TNBS. **(B**, **C)** Fibrotic changes histopathologically of mice from different groups were determined by Masson’s trichrome staining and EVG staining assays, and thalidomide inhibited fibrosis in intestinal tissue. (100×).

Severity of colonic inflammation was evaluated by HE staining assay. As shown in **Figure 3A**, colonic tissues from the sham-1 and sham-2 groups was almost normal with little inflammatory infiltration necrosis and edema. TNBS caused significant elevation in inflammatory response characterized by thickening of the mucosa, inflammatory cell infiltration with necrotic foci, extensive destruction of mucosal epithelium, loss of mucus-secreting cells, submucosal edema, necrosis and ulcer on the mucosal surface. The colonic tissues from thalidomide in modeling or after modeling groups exhibited a downward trend in the inflammatory infiltration. Secondly, Masson’s trichrome staining and EVG staining assays were performed to investigate the fibrotic changes histopathologically. The results of **Figure 3B** showed that a few inflammatory cells were scattered in the interstitium occasionally, and no mucosal ulcer was found in the sham-1 and sham-2 groups, while collagen infiltration in colonic tissues was deeper in model group. The colonic tissues from thalidomide in modeling or after modeling groups exhibited a downward trend in the collagen infiltration. Moreover, the data of EVG staining (**Figure 3C**, elastic fibers were blue-black) demonstrated that elastic fibers increased in model group compared with sham-1 and sham-2 groups, while thalidomide treatment in modeling or after modeling could reverse the established fibrosis.

### Effects of Thalidomide on the Production of Pro-Inflammatory and Anti-Inflammatory Cytokines in Mice Induced With TNBS

**Figure 4:**
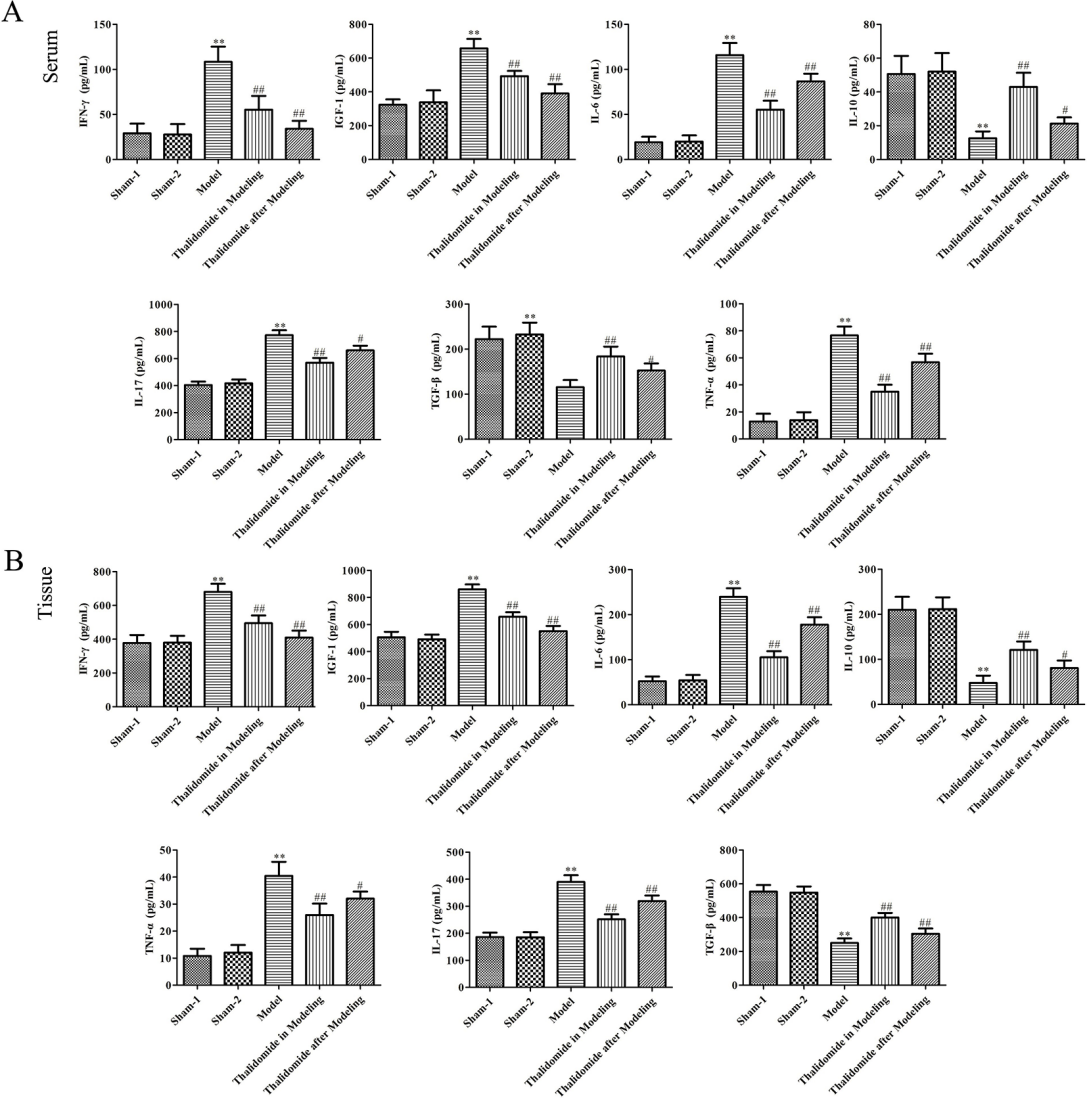
Effects of thalidomide on the production of inflammatory cytokines in mice induced with TNBS. Thalidomide treatment could obviously inhibit production of IFN-γ, IGF-1, IL-6, IL-17 and TNF-α, and promote the production of IL-10 and TGF-β in serum **(A)** and colonic tissues of mice from different groups **(B)**. The results were expressed as the mean ± SD. **P < 0.01 compared with sham-1 and sham-2 groups, ^#^ *P < *0.05, ^##^ *P < *0.01 compared with the model group.

To investigate the effect of thalidomide on the production of pro-inflammatory and anti-inflammatory cytokines in mice induced with TNBS, the serum and colonic tissues were collected. As described in **Figures 4A, B**, TNBS could significantly elevate the production of IFN-γ, IGF-1, IL-6, IL-17 and TNF-α, and decrease the secretion of IL-10 and TGF-β. However, thalidomide treatment could obviously inhibit production of IFN-γ, IGF-1, IL-6, IL-17 and TNF-α and promote the production of IL-10 and TGF-β both in modeling and after modeling.

### Effects of Thalidomide on mRNA and Protein Expression Levels Related to Fibrosis in Mice Induced With TNBS

**Figure 5:**
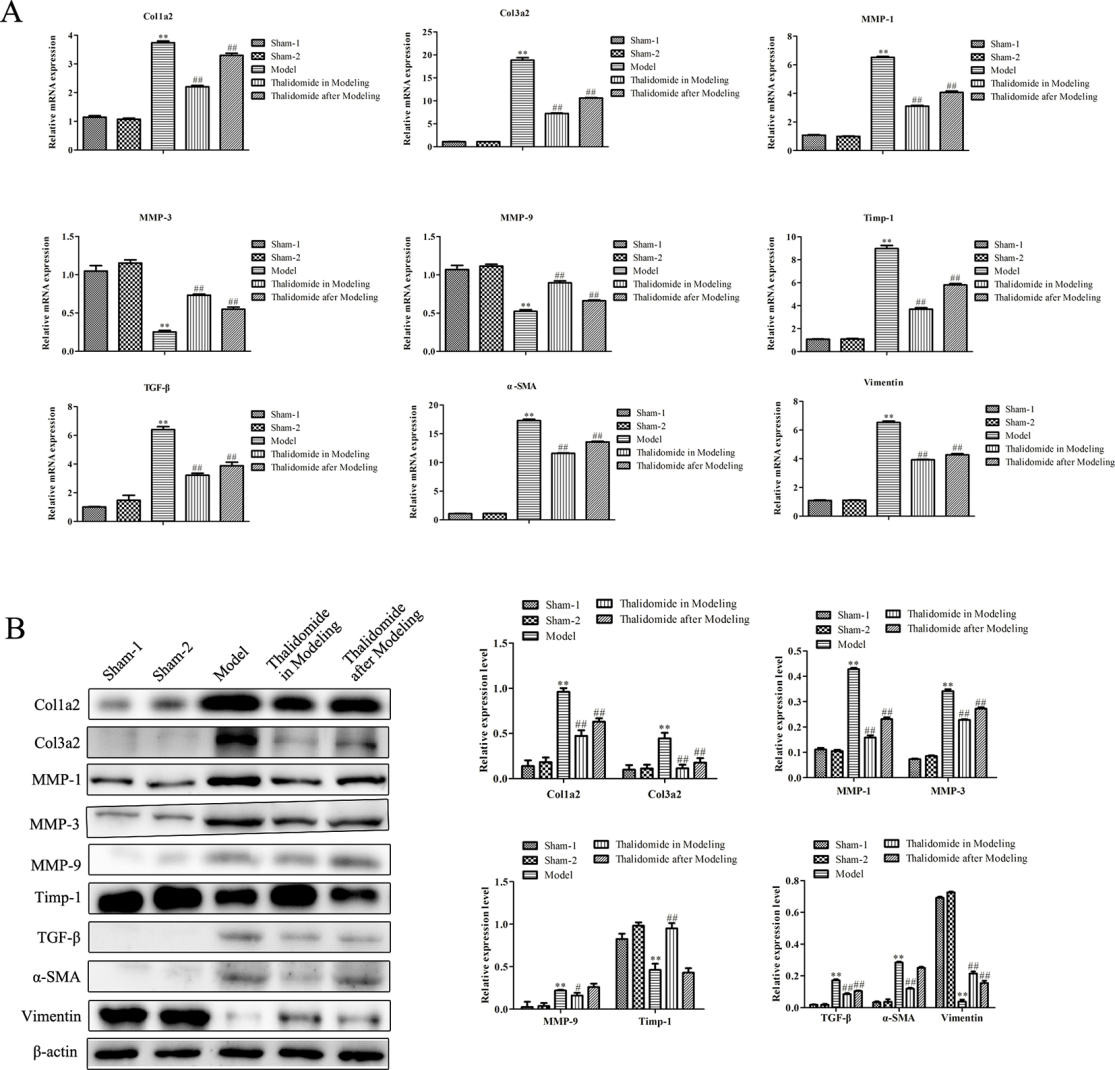
Effects of thalidomide on mRNA and protein expression related to fibrosis in mice induced with TNBS. **(A)** QRT-PCR assay was performed to evaluate the expression levels of fibrosis-related mRNA in mice from different groups. **(B)** Western blot assay was performed to evaluate the expression levels of fibrosis-related proteins in mice from different groups. **P < 0.01 compared with the sham-1 and sham-2 groups; ^#^ *P < *0.05, ^##^ *P < *0.01 compared with the model group.

**Figure 6:**
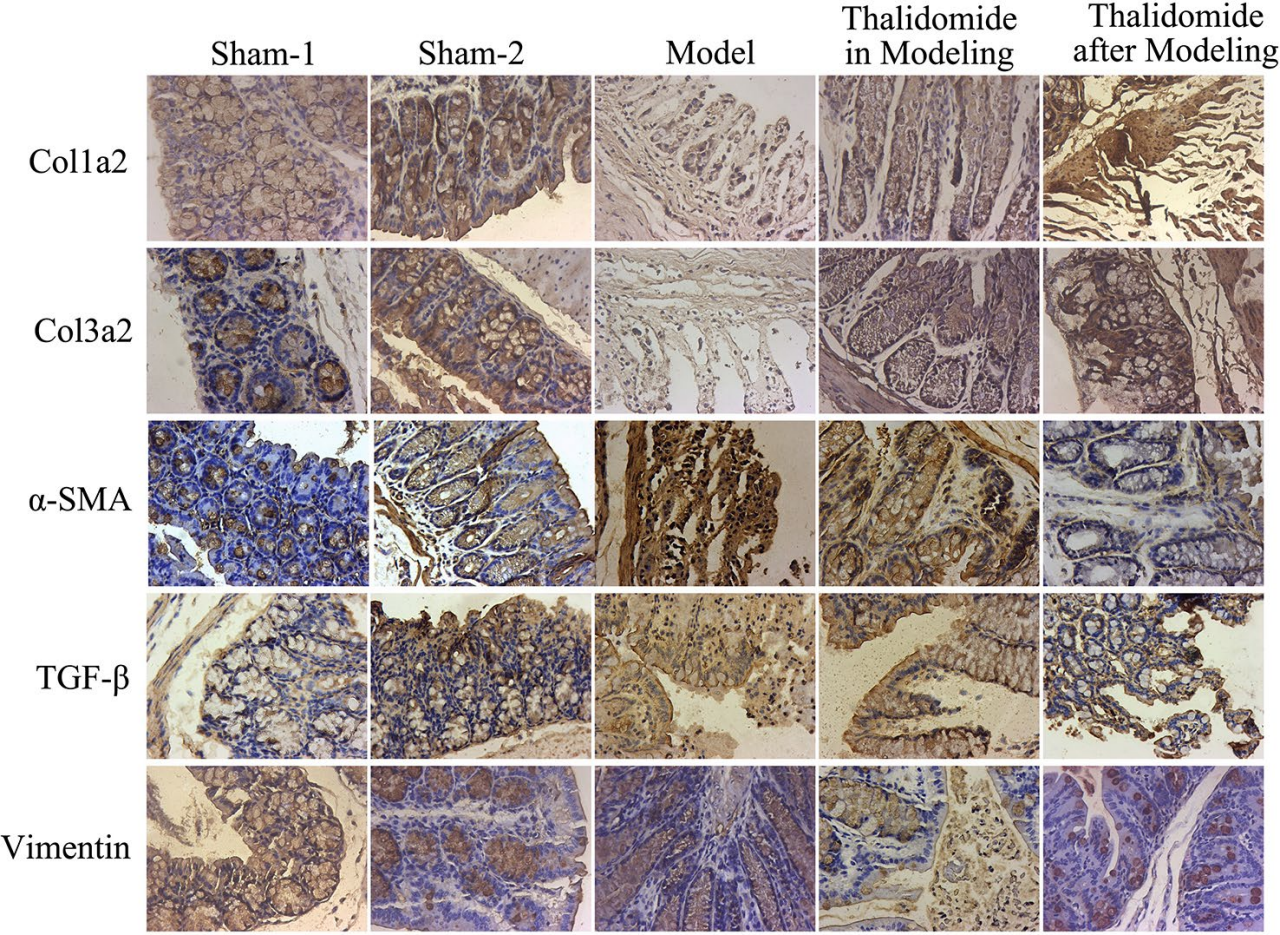
Immunohistochemical assay was performed to evaluate the expression levels of fibrosis-related proteins in mice from different groups. TNBS induction could significantly increase the expressions of col1a2, col3a2, TGF-β and α-SMA while decrease the expression of Vimentin, and thalidomide treatment could reverse the phenomenon.

Intestinal fibrosis is thought to be a common phenomenon in inflammatory bowel disease, and often causes serious complications in CD, eventually leading to intestinal stenosis. QRT-PCR and western blot assays were performed to evaluate the expression levels of fibrosis-related mRNA in serum and proteins in intestinal tissue. As shown in **Figure 5B**, compared with sham-1 and sham-2 groups, protein levels of col1a2, col3a2, MMP-1, MMP-3, MMP-9, TGF-β and α-SMA in intestinal tissues were obviously up-regulated while TIMP-1 and Vimentin were significantly down-regulated in model group, and thalidomide treatment in modeling or after modeling could obviously reverse the phenomenon. However, results from serum sample test showed that MMP3, MMP9 and Vimentin were inconsistent with this, **Figure 5A**. Furthermore, we also performed immunohistochemisty to evaluate the expression levels of col1a2, col3a2, α-SMA, TGF-β and Vimentin in intestinal tract. As expected, compared with the sham-1 and sham-2 groups, TNBS treatment could significantly increase the expressions of col1a2, col3a2, TGF-β and α-SMA while decrease the expression of Vimentin, and thalidomide treatment in modeling or after modeling could obviously reverse the phenomenon (**Figure 6**).

## Discussion

In recent years, the incidence of CD has been increasing, and the etiology of CD may be the complex interaction between genetic susceptibility, environmental factors and intestinal flora, which results in the imbalance of innate and adaptive immune responses (Geremia et al., 2014). The choice of treatment options for CD depends on the severity of the disease and the patient’s response to previous treatment (Boland and Vermeire, 2017). The traditional treatment of CD mainly includes aminosalicylic acid preparation, glucocorticoids, immunosuppressive agents, anti-TNF-α monoclonal antibody and the curative effect varies from person to person. However, some patients have no response or secondary with allergy, infection, tuberculosis or cancer. Therefore, refractory CD is still important problems facing clinicians.

Thalidomide has been proved to be effective for the treatment of refractory CD (Hu et al., 2016; Simon et al., 2016; Wang et al., 2017; He et al., 2017; Zhu et al., 2017; Lazzerini et al., 2017). Lazzerini et al. analyzed long term results of children treated with thalidomide (administered at a daily dosage of 50 mg, 100 mg, or 150 mg for patents <30 kg, 30-60 kg, and >60 kg), 54.3% maintained clinical remission, 41.4% achieved mucosal healing, and 28.6% achieved histologic healing (Lazzerini et al., 2017). He et al. reported 47 adults with active CD, who refractory to conventional therapies, treated with low-dose thalidomide (50-100 mg), clinical remission rate was 53.2% at week 24. Among 32 patients who underwent ileocolonoscopy at week 24, the rate of endoscopic response and complete endoscopic remission were 68.4% and 43.8% (He et al., 2017). However, the mechanisms of action of thalidomide are not yet entirely clear, its benefit has primarily been ascribed to its roles as an anti-TNF-alpha agent and reduce TNF-α, IL-6 and IL-1β production in colon homogenate of experimental colitis (Fakhoury et al., 2014). The present study was designed to further explore the effects and possible mechanisms of thalidomide on the prevention and treatment of experimental mice with CD. As expected, treatment with thalidomide in modeling or after modeling could increase the body weight of the mice induced by TNBS and protect the colon against TNBS-induced injury.

With further study of the pathogenesis of CD and pharmacological mechanisms of thalidomide, its therapeutic effects on CD have been paid more attention. The imbalance of Th17/Treg and Th1/Treg response is the key link in the pathogenesis of CD (Brand, 2009; Ramos et al., 2017). Th17 cells mainly participate in and promote the occurrence and development of autoimmunity, while secreting many pro-inflammatory cytokines, including IL-17, TNF-α, INF-γ, IL-1β, IL-6, IL-8, IL-21, IL-22, GM-CSF, as well as adhesion molecules ICAM-1, VCAM-1, which can induce and amplify inflammation (Bsat et al., 2019). In addition, Th1 cells can secrete IFN-γ, TNF-α and IL-2, resulting in inflammation and intestinal mucosal damage (Bsat et al., 2019). Previous studies have shown that the ratio of Th17 and Th1 cells in peripheral blood and intestinal mucosa was increased significantly in active CD while Treg cells were obviously decreased. In our study, we found that thalidomide treatment in modeling or after modeling could reduce the overexpression of Th cell (Th1 and Th17) cytokines including IFN-γ, IGF-1, IL-6, IL-17 and TNF-α in serum and intestinal tissues in TNBS-induced colitis model. Meanwhile, thalidomide could increase the expression of Treg cell cytokines such as IL-10 and TGF-β, indicating that adjusting the imbalance of Th17/Treg cells and restoring the body’s immune balance may be possible mechanisms of thalidomide in the treatment of CD. Furthermore, colonic tissues from thalidomide in modeling or after modeling groups exhibited a downward trend in the inflammatory infiltration of CD mice induced by TNBS.

In the development and progression of CD, continuously destroyed intestinal tissues, activated intestinal stromal cells and excessive interstitial extracellular matrix lead to intestinal fibrosis (Sahebally et al., 2013). Intestinal wall fibrosis in CD is mainly due to the imbalance of deposition and degradation of ECM, matrix metalloproteinase (MMPs), as the endopeptidases which are involved in the degradation of ECM, whose activity is regulated by matrix metalloproteinase inhibitor (TIMPs). It has been found that excessive deposition of ECM is a necessary condition for intestinal wall fibrosis and stricture (Latella et al., 2015). The collagen contents in fibrotic intestinal segments are significantly higher than those in control group, and the expression levels of collagens are positively correlated with the severity of intestinal inflammatory reaction at the same time (Scheibe et al., 2019). TGF-β is the key cytokine of fibrosis in most tissues and closely related to the development of fibrosis and the synthesis of ECM (Dong et al., 2016). TGF-β exhibits crucial effects on the regulation of fibrosis and wound healing, which directly or indirectly promotes fibrosis, including the migration and activation of ECM-producing cells and synthesis of ECM proteins (Levine et al., 1993). Moreover, many cytokines regulate ECM degradation by affecting the expression of MMPs and TIMPs (Mäkitalo et al., 2009; Piechota-Polanczyk et al., 2017). Some studies have indicated that thalidomide-mediated suppression of fibro-proliferation might contribute to the anti-fibrotic effect against pulmonary fibrosis (Horton et al., 2012; Dong et al., 2017), but there is no study on anti-fibrosis of thalidomide in treatment of CD currently. In the present study, the results demonstrated that thalidomide treatment in modeling or after modeling could reverse the established fibrosis and impeded the accumulation infiltration of CD mice induced with TNBS by Masson’s trichrome staining and EVG staining assays. Thalidomide treatment in modeling or after modeling down-regulated the expression levels of col1a2, col3a2, MMP-1, MMP-3, MMP-9, TGF-β, α-SMA, and up-regulated the expression levels of TIMP-1, indicating that thalidomide exhibited positive effects on treatment of CD accomplished by regulation of fibrosis.

In conclusion, the results of the present study demonstrated that thalidomide reduced the inflammatory response and inhibited damage of colon tissue in TNBS-induced mice colitis through restoring the imbalance of TH17/Treg cells and altering the balance of the pro- and anti-inflammatory cytokines. In addition, thalidomide inhibited intestinal fibrosis by regulating TIMP/MMPs protein balance, and degradation of ECM. The results will provide a theoretical basis for the treatment of CD with thalidomide in clinical.

## Data Availability Statement

All datasets generated for this study are included in the article/supplementary material.

## Ethics Statement

The animal study was reviewed and approved by the Institutional Animal Care and Use Committee of Jiangsu Province Hospital of Chinese Medicine and the Affiliated Hospital of Nanjing University of Chinese Medicine (2016NL-033-03).

## Author Contributions

BY, PZ and HC contributed to the conception and design of the study, acquisition, analysis and interpretation of data; HC, HX, LL and LQ performed the experiments; MX and YL collected the biopsy samples; YW performed pathological assessment; all authors drafted the article and made critical revisions, and approved the final version of the article to be published.

## Funding

This work was supported by Phase III Project and funded by the Priority Academic Program Development of Jiangsu Higher Education Institutions, the National Natural Science Foundation of China (81673973), the Natural Science Foundation of Jiangsu Province, China (BK2016157) and the Developing Program for High-level Academic Talent in Jiangsu Hospital of TCM (y2018rc16).

## Conflict of Interest

The authors declare that the research was conducted in the absence of any commercial or financial relationships that could be construed as a potential conflict of interest.
